# Feasibility and potential effectiveness of nurse-led video-coaching interventions for childhood, adolescent, and young adult cancer survivors: the REVIVER study

**DOI:** 10.1186/s12885-024-12430-3

**Published:** 2024-06-11

**Authors:** Eline Bouwman, Iridi Stollman, Joyce Wilbers, Joyce J. M. Claessens, Dick Johan van Spronsen, Annet Bongaerts, Dionne Breij, Nicole M. A. Blijlevens, Hans Knoop, Rosella P. M. G. Hermens, Jacqueline J. Loonen

**Affiliations:** 1 Department of Haematology, Centre of Expertise for Cancer Survivorship, Radboud university medical centre, Nijmegen, the Netherlands; 2 Department of Haematology, Radboud University Medical Centre, Nijmegen, the Netherlands; 3 Department of Medical Psychology, Amsterdam University Medical Centres, University of Amsterdam, Amsterdam Public Health Research Institute, Amsterdam, Netherlands; 4 Radboud University Medical Centre, IQ Health, Kapittelweg 54 (route 160, post 160), Nijmegen, HB 6500 the Netherlands

**Keywords:** eHealth, Interventions, Cognitive behaviour therapy, Motivational interviewing, Person-centred care, CAYA cancer survivors, Fatigue, Lifestyle, Empowerment

## Abstract

**Background:**

Childhood, adolescent, and young adult (CAYA) cancer survivors, at risk for late effects, including cancer-related fatigue, cardiovascular issues, and psychosocial challenges, may benefit from interventions stimulating behaviour adjustments. Three nurse-led eHealth interventions (REVIVER) delivered via video calls and elaborating on person-centred care, cognitive behaviour therapy and/or motivational interviewing were developed. These interventions target: 1) fatigue management, 2) healthier lifestyle behaviours, and 3) self-efficacy and self-management. This study aimed to assess the feasibility and potential effectiveness of the REVIVER interventions for CAYA cancer survivors and healthcare professionals.

**Methods:**

In a single-group mixed methods design, CAYA cancer survivors aged 16–54, more than five years post-treatment, were enrolled. Feasibility, assessed via Bowen's outcomes for feasibility studies, included *acceptability, practicality, integration and implementation, demand* and *adherence*. Qualitative data from semi-structured interviews and a focus group interview with survivors and healthcare professionals supplemented the evaluation. Paired sample t-tests assessed changes in self-reported quality of life, fatigue, lifestyle, self-management, and self-efficacy at baseline (T0), post-intervention (T1), and 6-month follow-up (T2).

**Results:**

The interventions and video consults were generally *acceptable, practical*, and successfully *integrated* and *implemented*. Success factors included the nurse consultant (i.e., communication, approach, and attitude) and the personalised approach. Barriers included sustainability concerns, technical issues, and short intervention duration. Regarding *demand*, 71.4%, 65.4%, and 100% of eligible CAYA cancer survivors engaged in the fatigue (*N* = 15), lifestyle (*N* = 17) and empowerment (*N* = 3) intervention, respectively, with 5, 5 and 2 participants interviewed, correspondingly. Low interest (*demand*) in the empowerment intervention (*N* = 3) and dropout rates of one-third for both fatigue and empowerment interventions were noted (*adherence*). Improvements in quality of life, fatigue (fatigue intervention), lifestyle (lifestyle intervention), self-efficacy, and self-management were evident among survivors who completed the fatigue and lifestyle interventions, with medium and large effect sizes observed immediately after the intervention and six months post-intervention.

**Conclusions:**

Our study demonstrates the feasibility of nurse-led video coaching (REVIVER interventions) despite lower demand for the empowerment intervention and lower adherence to the fatigue and empowerment interventions. The medium and high effect sizes found for those who completed the interventions hold potential clinical significance for future studies investigating the effectiveness of the REVIVER interventions.

**Supplementary Information:**

The online version contains supplementary material available at 10.1186/s12885-024-12430-3.

## Background

Over the last four decades, advances in treatments for childhood, adolescent and young adult (CAYA) cancer, defined as having received the diagnosis of cancer before the age of 39, have led to a dramatically expanded population of CAYA cancer survivors [1–5]. However, a distressing consequence of successful cancer treatment is that even years after treatment, CAYA cancer survivors are subject to an increased risk of developing adverse health conditions, i.e., late effects [6–13].

Late effects may cause chronic morbidity and premature mortality in CAYA cancer survivors and can negatively impact multiple dimensions of health-related quality of life (HRQOL) [14–17]. One notable example of such a late effect that may affect HRQOL negatively is cancer-related fatigue (CRF), defined as “a distressing, persistent, subjective sense of tiredness or exhaustion that is not proportional to recent activity and even interferes with usual functioning” [16–18]. The high prevalence of cancer-related fatigue (CRF) among adult survivors of childhood cancer, reaching 26.1% compared to 14.1% in their sibling controls, emphasises the crucial need to address and prioritise CRF in cancer survivorship care [19]. In addition, cardiovascular diseases, primarily attributed to the use of anthracyclines, mitoxantrone, and/or chest-directed radiation as treatment modalities, are frequently reported in CAYA cancer survivors as well [15, 20–22]. Examples of these serious cardiac late effects may include heart failure, as well as coronary heart-, valvular heart- and pericardial diseases. While treatments may predispose CAYA cancer survivors to these conditions, Chen et al. demonstrated that traditional cardiovascular risk factors, including hypertension, dyslipidaemia, and diabetes, are also noteworthy contributors in this population [23]. Besides the physical late effects, the overall impact of cancer diagnosis, treatment, and enduring late effects can negatively affect the psychosocial functioning of CAYA cancer survivors, leading to conditions such as depression, anxiety, and psychological distress, ultimately impacting HRQOL [24–26]. Research by Michel et al. has shown a range of psychological distress in CAYA cancer survivors, from 6% to as high as 30% [27]. Given the potential hypothesis that diminished psychosocial functioning may influence self-efficacy and self-management capabilities, it becomes imperative to address and intervene in psychosocial health within the framework of cancer survivorship care.

CAYA cancer survivors, despite their susceptibility to late effects, can mitigate some risks by adopting healthier behaviours. Gielissen et al. conducted a randomised controlled trial showing significant improvements in fatigue severity (54% vs. 4%) and functional impairment (50% vs. 18%) with cognitive behaviour therapy compared to a waiting list [28]. Additionally, Abrahams et al. found that Internet-based cognitive behaviour therapy effectively reduced severe fatigue in breast cancer survivors compared to standard care [29]. Moreover, other studies highlight the positive impacts of healthy behaviours on various health outcomes in cancer survivors, such as a 20% reduced risk of health-related mortality shown by Dixon et al. independent of traditional cardiovascular risk factors [30–33]. Lastly, Buffart et al. found that, among cancer survivors (≥ three months post-treatment, 57% breast cancer survivors), physical exercise has been associated with improved quality of life, mediated by increased physical activity, general self-efficacy, and mastery [34]. Long-term follow-up care for cancer survivors is vital for promoting health behaviours. However, challenges like limited financial and human resources and a growing survivor population require sustainable and cost-effective solutions. Electronic health (eHealth) interventions, accessible from home without intensive coaching, emerge as effective alternatives for delivering care with constrained resources [35]. These interventions reduce the need for clinic visits and minimise disruption to survivors' daily activities, as demonstrated in various studies [29, 36, 37]. Recently, eHealth interventions, delivered via 3–6 video calling sessions by a trained nurse consultant, were developed for survivors of CAYA cancer to reduce CRF and/or promote healthy lifestyle behaviours or to empower survivors by stimulating self-efficacy and self-management [38]. These so-called REVIVER interventions were developed patient-driven and based on person-centred care, cognitive behaviour therapy (CBT) and/or motivational interviewing. However, the REVIVER interventions have not yet been evaluated regarding feasibility and potential effectiveness. Bowen’s framework can provide valuable guidance in designing feasibility studies based on key outcomes that can determine feasibility [39]. Therefore, in order to evaluate and implement the REVIVER interventions on a broader scale, the primary aim of this study was to determine the feasibility of the REVIVER interventions for both CAYA cancer survivors and healthcare professionals (HCPs) in terms of the following outcomes of Bowen’s areas of focus for feasibility studies [39]:

- *Acceptability: To what extent are the content and delivery of the REVIVER interventions judged appropriate and satisfying by CAYA cancer survivors and healthcare professionals?*
- *Practicality: To what extent can the REVIVER intervention activities be carried out with CAYA cancer survivors and healthcare professionals (i.e., by using a video calling application as a mode of delivery and perceived effect intervention activities on survivors)?*
- *Integration and implementation: To what extent can the REVIVER interventions be integrated into the current care system and successfully delivered to CAYA cancer survivors (i.e., barriers and facilitators of the interventions and implementation, success and failure of executions, and sustainability of the interventions)?*
- *Demand*: *To what extent are the REVIVER interventions likely used by CAYA cancer survivors (i.e., participation rate and expressed interest)?*
- *Adherence: How compliant are CAYA cancer survivors with the REVIVER intervention activities (i.e., dropout rate, and adherence to sessions)?*

Our secondary aim was to gain insight into the potential effectiveness of the REVIVER interventions in CAYA cancer survivors regarding HRQOL, fatigue, lifestyle behaviours, self-efficacy and self-management.

## Methods

The protocol paper by Bouwman et al. [38] detailed the content of the REVIVER interventions and methods applied in the REVIVER study. In short, the REVIVER interventions are designed for CAYA survivors to receive coaching to cope with the direct or indirect late effects of cancer. The three interventions, elaborating on person-centred care principles, are aimed at the improvement of (i) symptoms of Cancer Related Fatigue (CRF), (ii) self-efficacy and self-management or (iii) lifestyle. They consist of an intake and 3 to 6 screen-to-screen video-coaching sessions delivered within three months. A reflection session will follow after a 6-month period in which the survivor can actively work on his or her goals set during the coaching sessions. The interventions are led by a trained nurse who applies cognitive behaviour therapy (CBT), motivational interviewing (MI) or a combination of both to help survivors overcome their late effects.

### Design

The REVIVER study involved a mixed methods research approach, combining qualitative (i.e., semi-structured video calling interviews and a focus group) and quantitative measures (i.e., questionnaires) to assess feasibility and potential effectiveness. A single-arm pre- and post-test design with three measurement points was used to assess potential effectiveness. It was applied in a real-world setting of the survivors with their usual care providers (i.e., nurse consultants).

### Setting

Recruitment was conducted during clinic visits at a survivorship care clinic affiliated with a university hospital in an urban area in the Netherlands. The interventions were delivered remotely by screen-to-screen video calling with a nurse consultant based at the clinic. Online data collection for survivors took place at baseline (T0), directly after the intervention (T1), and six months post-intervention (T2). Additionally, a subset of survivors was approached for individual interviews between December 2021 and May 2022. Healthcare professionals participated in a focus group in October 2022.

### Study population

CAYA cancer survivors were primarily enrolled through referral by late-effect physicians and nurse consultants during regular medical follow-up consultations at the survivorship care clinic. Pre-send questionnaires and anamnesis assessed intervention applicability. To participate, CAYA cancer survivors (diagnosed with any cancer under the age of 39), currently aged 16–54 years, in complete cancer remission, needed a referral from HCPs, basic Dutch proficiency, and access to an Internet-enabled device (e.g., smartphone or tablet). Exclusions applied to those with fatigue from an underlying medical condition (fatigue intervention), complex endocrine disorders explaining overweight (lifestyle intervention), and/or cognitive or psychosocial issues (e.g., depression) affecting proper participation in the intervention. Lastly, survivors concurrently engaged in another intervention were excluded. Additionally, to ensure data triangulation with input from healthcare professionals, all HCPs involved in the referring process or coaching aspects of the REVIVER interventions (i.e., nurse consultants, physicians, and psychologists) were recruited for a focus group on their experiences with the interventions.

### Sample size

#### Feasibility evaluation—qualitative assessments

A total of 15 participants (5 per intervention) who completed the REVIVER interventions were selected for individual interviews to represent each intervention. The chosen number of participants was sufficient for the study's narrow and specific aim, providing substantial information power and ensuring code saturation [40, 41].

#### Potential effectiveness evaluation—quantitative assessments

As outlined in the study protocol paper, recruiting 60 participants (20 per intervention type) was considered feasible to gain insights into potential effectiveness through a feasibility study [38].

### Procedures

#### Feasibility evaluation – qualitative assessments

After the interventions, semi-structured interviews were conducted with CAYA cancer survivors. Likewise, a focus group interview was performed with all HCPs involved. To ensure credibility using investigator triangulation, two researchers (EB and DB), experienced and trained in qualitative research, facilitated the interviews and the focus group. Additionally, a note-taker (IS) was present to take field notes of key points raised during the focus group. To assess feasibility qualitatively regarding acceptability, practicality, and integration and implementation, the interview guides of the interviews and focus group covered questions on satisfaction and appropriateness of the delivery, content and mode of delivery (video calling) of the three interventions and perceived barriers and facilitators. It concluded with ratings on the interventions and the likelihood of recommending them to other survivors (survivors only). The interviews and focus group were audio recorded, transcribed verbatim, and anonymised. On average, the interviews lasted approximately 29 min each. In addition, one focus group with all involved HCPs was conducted, lasting 35 min on average.

#### Feasibility evaluation – quantitative assessments

Feasibility of the REVIVER interventions in terms of demand and adherence was assessed quantitatively with nurse consultants' reports on the duration and frequency of the sessions and reported barriers and facilitators*.* Integration and implementation were also assessed quantitatively by reported barriers and facilitators in the nurse consultants’ reports.

#### Potential effectiveness evaluation – quantitative assessments

CAYA survivors were provided with questionnaires on quality of life, fatigue, lifestyle behaviours, self-efficacy, and self-management (see **Outcome measures**) at three time points: baseline (T0), post-intervention (T1), and 6-month follow-up (T2). Sociodemographic and clinical information was extracted from medical records.

### Outcome measures

Predefined criteria for the success of the REVIVER interventions are displayed in Supplementary Table 1.

#### Feasibility evaluation – qualitative assessments

##### Acceptability

Acceptability, defined as the appropriateness of the intervention activities and satisfaction with both the content and delivery of the intervention, was evaluated by conducting interviews and a focus group.

##### Practicality

Interviews and a focus group evaluated the ability to carry out the intervention activities with CAYA cancer survivors and healthcare professionals (e.g., using a video calling application as a mode of delivery and their perceived effects on survivors) as indicators of practicality.

##### Integration and implementation

Integration refers to how the interventions can be integrated into existing healthcare systems, while implementation refers to how they can be implemented and successfully delivered in a healthcare system. In this study, integration and implementation were combined as feasibility outcome measures as they are closely related. Using the results of the interviews and focus group, integration and implementation were both measured by barriers and facilitators of intervention, success and failure of execution, barriers and facilitators affecting implementation ease, quality of implementation and perceived sustainability of the intervention.

#### Feasibility evaluation – quantitative assessments

##### Integration and implementation

Successful integration and implementation of the REVIVER interventions were also assessed with barriers and facilitators, as reported by nurse consultants. The results regarding “Integration and implementation” are combined in the Results section for readability.

##### Demand

Demand (i.e., the likelihood that the interventions will be used by CAYA cancer survivors) was determined by the participation rate, which was defined as the number of survivors participating in the intervention vs. the number of survivors invited to participate and assessed using the nurse consultants' reports. The expressed interest in the study (i.e., the number of survivors invited to participate) was also determined as an indicator of demand.

##### Adherence

Adherence (i.e., compliance) was assessed through two measures. First, the dropout rate, representing participants who discontinued the intervention after completing T0, was calculated. Additionally, the percentage of participants adhering to the agreed-upon session schedule with the nurse consultant was evaluated. Both aspects of adherence were determined through the analysis of reports.

#### Potential effectiveness evaluation – quantitative assessments

##### Health-related quality of life (HRQOL)

The primary outcome measure to assess the potential effectiveness of the REVIVER interventions was HRQOL, as assessed with the disease-specific EORTC quality of life questionnaire (QLQ-C30) (Cronbach’s alpha = 0.95) [42–44]. In this questionnaire, HRQOL is assessed by functional scales (i.e., physical-, role-, emotional-, cognitive-, and social functioning) and several symptom scales (no symptom scale was used for this study) [43, 44].

##### Fatigue

The CIS20 questionnaire assessed fatigue. It explores four dimensions of fatigue, including severity, concentration problems, reduced motivation, and reduced physical activity (Cronbach’s alpha ranging from 0.84 to 0.95) [45, 46].

##### Lifestyle

To monitor changes in lifestyle behaviours over time, the Dutch “Leefstijlvragenlijst” was utilised, comprising of a compilation of existing validated questionnaires: the Fagerström Test for Nicotine Dependence (Cronbach’s alpha = 0.64), the short version of the International Physical Activity Questionnaire (IPAQ) (Cronbach’s alpha = 0.60), a questionnaire on eating habits and the Alcohol Use Disorders Identification Tests (AUDIT) (Cronbach’s alpha approximately 0.80) [47–52]. Physical activity was further evaluated using the SQUASH questionnaire, which included four domains: commuting activities, physical activity at work or school, household activities, and spare time [53]. The SQUASH questionnaire is reliable and reasonable, with a Spearman correlation coefficient for overall reproducibility of 0.58 in Dutch subjects.

##### Self-efficacy

Self-efficacy was determined using the General Self-Efficacy (GSE) scale, a 10-item questionnaire on how someone thinks or acts in particular situations (Cronbach’s alpha = 0.85) [54].

##### Self-management

Self-management was assessed using the Self-Management Screening (SeMaS) questionnaire, which measures someone's capability to self-manage chronic diseases and difficult situations (Cronbach's alpha for coping = 0.70; Cronbach's alpha for self-efficacy = 0.80) [55].

#### Fidelity

The nurse consultants' adherence to the intervention protocol was evaluated in two ways. First, the content of the intervention was assessed using the nurse consultants' reports, interviews with participants, and the focus group with HCPs to determine whether each of the intervention components was implemented as planned. Second, the frequency and duration of the intervention were evaluated using the nurse consultants’ reports.

### Analysis

#### Feasibility evaluation—qualitative assessments

**Fig. 1:**
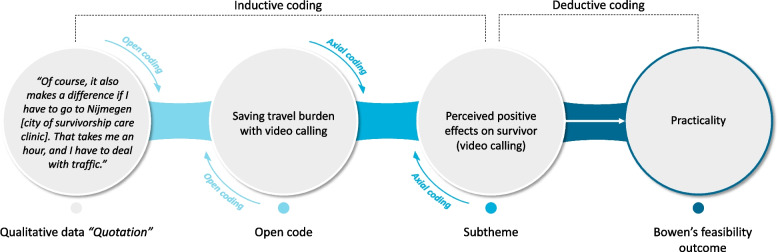
Illustrative example of the coding process

Atlas.ti 8.3.20 for Windows, a qualitative data analysis software program, facilitated the analysis. Thematic analysis was applied, involving two researchers (EB and IS) independently coding the transcripts on a sentence level. This process of investigator triangulation was performed in order to increase the trustworthiness of the data and its interpretation. Initially, an inductive-driven approach was conducted by open coding, with the created codes discussed after each transcript to reach a consensus (Fig. 1). Next, axial coding categorised these codes into barriers and facilitators and grouped the codes into subthemes [56]. As shown in Fig. 1, this process is bidirectional, facilitating ongoing research reflectivity on the data. Lastly, these subthemes were deductively mapped to the outcomes of Bowen’s focus areas for feasibility assessment in studies [39]. Any discrepancies in the analysis between EB and IS were discussed until consensus was reached. Reaching consensus was achieved by considering the original research question and thoroughly studying the quotation’s context. A third independent person was consulted if discrepancies could not be solved.

#### Feasibility evaluation – quantitative assessments

The quantitative analysis of demand, adherence, integration, and implementation was conducted as described in the section “ Outcome measures.”

#### Potential effectiveness evaluation – quantitative assessments

Quantitative outcome measures analysis was conducted using IBM software SPSS (v27). Descriptive statistics were employed, including raw numbers and percentages, means with standard deviations (SDs), and median with interquartile range. Paired t-tests were performed to compare means for primary and secondary outcome measures at baseline (T0), post-intervention (T1), and six months follow-up (T2) within the same participant when there was at least one set of data available (i.e., T0-T1 and T0-T2). Cohen’s effect sizes (*d*) were calculated to quantify observed differences over time, with values around 0.2, 0.5, or ≥ 0.8 indicating small, medium, or large effect sizes, respectively, based on Cohen’s guidelines [57]. A significance level of *P* < 0.05 was considered as a potential effect.

## Results

### Sample characteristics

In total, 12 survivors participated in the fatigue intervention, with 5 participating in interviews. For the lifestyle intervention, 17 participants agreed to take part, and 5 of them were interviewed. Finally, the empowerment intervention involved 3 participants, with 2 being interviewed. Following initial sample analysis, code saturation was evaluated by reflection on the repetition of observed patterns and codes. Code saturation had been achieved as no new codes emerged from the data. However, limited empowerment intervention participants (*n* = 2) constrained code saturation, preventing additional interviews. Table 1 shows the baseline characteristics of the study participants. Over half of the fatigue and lifestyle intervention participants were female against all of the participants in the empowerment group. Median survivor ages were 32 and 30 for the fatigue and lifestyle intervention, respectively, compared to 26 in the empowerment intervention group. Fatigue intervention participants were more likely to have completed a middle level of education, while other interventions had notably higher educational levels. The median age at cancer diagnosis ranged from 10 (empowerment intervention) to 15 (fatigue intervention), with CNS and germ cell tumours being the most prevalent diagnoses in the fatigue and lifestyle interventions, respectively. Empowerment intervention participants were survivors of Hodgkin lymphoma, a CNS and germ cell tumour. Chemotherapy dominated as the primary treatment modality, and comorbidities, excluding fatigue, were rare. Overweight and obesity were highly prevalent in the lifestyle intervention (29.4% and 64.7%, respectively) and fatigue intervention (33.3% and 26.7%, respectively) participants. Among the focus group of HCPs (*N* = 6), 5 females were included (83.3%) with a median age of 52 years and 25 years of working experience (5 years in cancer survivorship care).

**Table 1:** Baseline characteristics participants REVIVER study (n (%))

	Fatigue (*N* = 15)	Lifestyle (*N* = 17)	Empowerment (*N* = 3)	Focus group HCPs (*N* = 6)
Female gender	8 (53.3%)	9 (52.9%)	3 (100%)	5 (83.3%)
Age at inclusion, years^a^	32 (16)	30 (12)	26	52 (18)
Educational level^b^				
Low	0 (0%)	1 (5.9%)	0 (0%)	
Middle	11 (73.3%)	7 (41.2%)	0 (0%)	
High	4 (26.7%)	9 (52.9%)	3 (100%)	
Employment status				
Unemployed	3 (18.8%)	1 (5.9%)	0 (0%)	
Employed	11 (68.8%)	15 (88.2%)	2 (66.6%)	
Student	2 (12.5%)	1 (5.9%)	1 (33.3%)	
Age at diagnosis, years^a^	15 (8)	13 (11)	10^c^	
Category of childhood cancer diagnosis				
Leukaemia	2 (13.3%)	1 (5.8%)	0 (0%)	
Hodgkin lymphoma	1 (2.9%)	4 (23.5%)	1 (33.3%)	
Non-Hodgkin lymphoma	1 (2.9%)	1 (5.8%)	0 (0%)	
CNS tumours	4 (26.7%)	0 (0%)	1 (33.3%)	
Neuroblastoma	1 (6.7%)	0 (0%)	0 (0%)	
Renal tumours	0 (0%)	1 (5.8%)	0 (0%)	
Germ cell tumours	3 (20%)	5 (29.4%)	1 (33.3%)	
Bone tumours	3 (20%)	1 (5.8%)	0 (0%)	
Soft tissue tumours	0 (0%)	2 (11.8%)	0 (0%)	
Other and unspecified	0 (0%)	2 (11.8%)	0 (0%)	
Category of cancer treatment				
Surgery only	0 (0%)	1 (5.9%)	1 (33.3%)	
Chemotherapy, no radiotherapy	7 (46.7%)	12 (70.6%)	2 (66.6%)	
Radiotherapy, no chemotherapy	2 (13.3%)	1 (5.9%)	0 (0%)	
Surgery and chemotherapy	3 (20%)	1 (5.9%)	0 (0%)	
Surgery and radiotherapy	1 (6.7%)	0 (0%)	0 (0%)	
Chemotherapy and radiotherapy	1 (6.7%)	1 (5.9%)	0 (0%)	
Stem cell transplantation and chemotherapy	0 (0%)	1 (5.9%)	0 (0%)	
Immunochemotherapy	1 (6.7%)	0 (0%)	0 (0%)	
Comorbidities^d^				
None	13 (86.7%)	16 (94.1%)	2 (66.6%)	
1	1 (6.7%)	0 (0%)	1 (33.3%)	
≥ 2	1 (6.7%)	1 (5.9%)	0 (0%)	
BMI				
Normal	6 (40%)	1 (5.9%)	3 (100%)	
Overweight	5 (33.3%)	5 (29.4%)	0 (0%)	
Obese	4 (26.7%)	11 (64.7%)	0 (0%)	
Work experience, years^a^				25 (26)
Work experience in cancer survivorship care, years^a^				5 (2)
Current profession				
Nurse consultant				2 (33.3%)
Internist/ haematologist/ neurologist				3 (50%)
Psychotherapist				1 (16.7%)
Function in REVIVER study				
Referrer				5 (83.3%)
Coach				1 (16.7%)
Previous experience with video calling				5 (83.3%)

^a^Median (IQR)

^b^Low: primary education, technical and vocational education and training, special education; Middle: preparatory secondary vocational education, secondary vocational education, higher general secondary education, pre-university education

^c^Not enough participants to determine the interquartile range

^d^Assessed with the core set of self-reported long-term physical outcomes of clinical relevance for childhood cancer survivors as developed by Streefkerk et al., excluding CRF [58]

They included 2 nurse consultants, 3 late-effect physicians, and 1 psychologist. This group also included 5 referrers and 1 coaching nurse consultant. All but one HCP had prior video-calling experience.

### Results feasibility evaluation

#### Feasibility evaluation – qualitative assessments

##### Acceptability

Acceptability findings from participant interviews and an HCP focus group in the REVIVER interventions are displayed in Tables 2, 3 and 4. Overall, survivors had a positive perception of all three interventions, recommending them to others and giving "moderately good" (empowerment) or "good" (fatigue and lifestyle) scores. For both the empowerment and lifestyle interventions, additional coaching or psychosocial help was frequently needed after the completion of the intervention. Notably, for the lifestyle intervention, this was not consistently perceived negatively as the intervention often served as a preparatory step towards more profound help, as illustrated by the following quote:

*"And what I needed for that [i.e., losing weight and increasing physical activity] was not so much someone who tells me what I should or should not eat, but someone who will convince me, who looks together with me and [asks]: “Okay, what actions can we take? […]. What could we do to make you cross that line?” (Female, 29 y, participant REVIVER lifestyle intervention).*

**Table 2:** Summary of CAYA cancer survivors’ perspectives regarding barriers of the REVIVER interventions

Area of focus and feasibility construct	Barrier	Fatigue	Lifestyle	Empowerment
**Acceptability**				
Satisfaction	• Preference face-to-face intervention in future	X		X
Perceived appropriateness intervention for survivors	• Second coaching trajectories/psychosocial help needed after intervention		X	X
	• Intervention superficial			X
	• Intervention not suitable			X
Expectations intervention	• Sceptical attitude beforehand	X	X	
	• Expectations not meeting reality (negative)	X	X	X
**Practicality**				
Ability of survivor to carry out intervention activities (after intervention)	• Relapse after intervention	X	X	
	• Difficulties with maintenance after intervention	X	X	
Ability of survivor to carry out intervention activities (during intervention)	• Technical problems video calling hindering intervention activities	X	X	X
	• Distraction in busy environments hindering intervention activities		X	
	• Personal circumstances negatively influencing outcomes intervention	X	X	X
	• Personal mindset negatively influencing outcomes intervention		X	
	• Lack of skills negatively influencing outcomes intervention		X	
Perceived effects on survivors (video calling)	• Negative experiences video calling	X	X	
Perceived effects on survivors (intervention)	• Exercises intervention not helpful	X		
	• Intervention not successful	X		X
	• Unclear effect intervention	X	X	
	• Uncomfortable feeling with personal intervention		X	X
**Integration/implementation**				
Barriers of intervention	• Study barrier for intervention	X		X
	• Duration between sessions too long		X	X
	• Duration intervention too short		X	
	• Duration sessions too short			X
	• More sessions needed		X	X
	• Tips not specific		X	X
Failure of execution	• Unclear information provision beforehand	X	X	X
Quality of implementation	• Qualifications nurse inadequate		X	
	• Overqualified nurse			X

**Table 3:** Summary of CAYA cancer survivors’ perspectives regarding facilitators of the REVIVER interventions

Area of focus and feasibility construct	Facilitator	Fatigue	Lifestyle	Empowerment
**Acceptability**				
Satisfaction	• Intervention recommended to other survivors	X	X	X
	• Overall rating intervention "moderately good"			X
	• Overall rating intervention "good"	X	X	
	• Positive attitude towards overall intervention	X	X	X
	• Positive attitude towards intake session	X	X	
	• Positive attitude towards coaching sessions	X	X	X
	• Positive attitude towards closing session	X	X	X
	• Preference for partial video calling sessions	X	X	
	• Choosing video calling interventions again in future	X	X	X
Perceived appropriateness intervention for survivors	• Video calling intervention suitable			X
Expectations intervention	• Expectations meeting reality (positive)	X	X	
	• Expectations not meeting reality (positive)	X	X	
	• No expectations prior intervention	X	X	X
**Practicality**				
Ability of survivor to carry out intervention activities (after intervention)	• Application tips/intervention good in daily life	X	X	X
	• Maintenance good habits after intervention • Sustainable changes after intervention	X	X	
Ability of survivor to carry out intervention activities (during intervention)	• Good internet connection facilitating intervention activities	X	X	
	• Technical problems solved for intervention activities	X	X	X
Perceived effects on survivors (video calling)	• Saving travel burden with video calling	X	X	X
	• Positive experiences video calling	X	X	X
Perceived effects on survivors (intervention)	• Goal completion		X	X
	• Intervention gives push in right direction		X	
	• Positive results indirectly related to intervention	X	X	
	• Intervention fulfilling needs survivor	X	X	X
	• Positive feelings with intervention		X	
	• Intervention creating health consciousness		X	X
	• Empowerment by intervention			X
	• Breaking up wrong routines with intervention	X	X	
	• Improvement lifestyle behaviours with intervention		X	
	• Physical health benefits with intervention	X	X	
**Integration/implementation**				
Success factors of intervention	• Sufficient amount of sessions	X	X	X
	• Appropriate duration sessions		X	
	• Appropriate duration between sessions	X	X	X
	• Completeness intervention	X	X	X
	• Intervention delivered by a nurse		X	
	• Good qualities of nurse	X	X	X
	• Good approach and attitude nurse	X	X	X
	• Good communication by nurse	X	X	X
	• Ability to share own story	X	X	X
	• Having personal conversations	X	X	X
	• Attention for survivor		X	
	• Personal contact with video calling	X		
	• Following intervention from location by choice		X	X
	• Flexible planning	X	X	X
	• Personalised approach intervention	X	X	X
	• No pressure/obligations with intervention	X	X	X
	• Small step approach	X	X	
	• Coaching aspect intervention	X	X	X
	• Accountability aspect intervention	X		
	• Provided advices/exercises	X	X	X
	• Broad intervention			X
	• Easiness intervention	X		
	• Closure intervention			X
	• Provision by late effects clinic			X
Success of execution	• Good information provision beforehand	X	X	X
	• Starting intervention unprejudiced		X	X
	• Good relationship with nurse	X	X	X
	• Good adherence survivor to intervention	X		
	• Motivated survivor facilitating intervention	X	X	
	• Success dependent on personal factors		X	
	• Prior knowledge survivor healthy behaviours helpful		X	
	• Appropriate goal/module set	X	X	
	• HCPs contributing to participation survivor	x	X	X
Quality of implementation	• Competent nurse	X		X

**Table 4:** Summary of HCPs’ perspectives regarding the feasibility of the REVIVER interventions

Area of focus and feasibility construct	Barrier	Facilitator
**Acceptability**		
Fit within organizational culture		• Video calling interventions part of vision organization
Satisfaction		• Positive attitude towards video calling
		• Overall rating intervention "good"
		• Overall rating intervention "moderately good"
		• Positively minded about interventions
Perceived appropriateness intervention for survivors	• Interventions not suitable for everyone	• Video calling suitable for delivering intervention
		• Intervention suitable for many survivors
Intent to continue use		• Openness towards video calling interventions in future
**Practicality**		
Ability of survivor to carry out intervention activities	• Unmotivated survivor hindering intervention activities	• Provision tools to sustain behaviour facilitating intervention activities
	• Technical problems video calling hindering intervention activities	• High applicability interventions facilitating intervention activities
		• Readiness survivor facilitating intervention activities
		• Motivated survivor facilitating intervention activities
Perceived effects on survivor (video calling)		• Saving travel burden for survivors with video calling
Perceived positive effects on survivor (intervention)		• Interventions perceived as helpful for survivors
	• Enthusiastic nurse helpful	• Enthusiastic nurse helpful
**Integration/implementation**		
Barriers/success factors of intervention	• Sustainability lifestyle intervention insufficient	• Open approach empowerment intervention
	• Lack of guidelines in lifestyle intervention	• Research setting of intervention
		• Aftercare after clinic visit
		• Hospital setting
		• Personalised interventions
		• Low threshold aspects intervention
		• Communication with survivors
		• Delivering intervention in own environment survivor
		• Flexibility with video calling
Failure/success of execution	• Difficulties contacting survivor hindering execution intervention	• High impact HCP on participation
	• Difficulties with survivors dropping out hindering execution intervention	• Clear referral criteria and procedures for inclusion intervention
	• Wrong timing intervention hindering intervention	• Good infrastructure to include survivors
		• Good procedures for handling timing issue with survivor by HCP
Quality of implementation	• Insufficient knowledge provision in lifestyle intervention	• Multidisciplinary meetings to discuss patients improving quality intervention
		• Nurse receiving supervision in coaching improving quality intervention
		• Own perceived capability of nurse good
		• "Learning on the job" facilitating delivering intervention
Barriers/ facilitators affecting implementation ease	• Inadequate feedback provision structure to referrers	• Support from others facilitating implementation
	• Lack of funding/resources for implementation	• Low costs of resources facilitating implementation • Good software video calling facilitating implementation
		• Sufficient feedback provision to referrers facilitating implementation
Perceived sustainability of intervention		
		• Feasible on long term to implement interventions

Video calling was generally deemed acceptable, and survivors indicated a willingness to opt for future video-calling interventions (Tables 2 and 3). The HCPs involved in the referring or coaching also expressed satisfaction with the three interventions (Table 4). The nurse consultant delivering the interventions found video calling to positively exceed her expectations regarding building a connection with survivors:

*And what surprised me very positively is that you have really good contact via video calls. That it really succeeds in building good contact […]. I think that is very positive. Initially, people think, " You have to be in one room [for delivering interventions] […]." But I noticed I don't feel it as an obstacle to do that as a coach, so that surprised me very positively.“ (Female, 60 y, nurse consultant/coach REVIVER interventions).*

Video calling also aligns seamlessly with the hospital's overarching vision and is therefore encouraged by the organisation. For this reason, HCPs exhibited openness towards video calling interventions in the future.

##### Practicality

Regarding the practicality of the REVIVER interventions, findings yielded mixed responses concerning the sustainability of the fatigue and lifestyle interventions (Tables 2 and 3). While most survivors maintained good health habits and experienced sustainable changes in fatigue long-term, some experienced setbacks after some time, reverting to old patterns. Generally, the tips and interventions were perceived as applicable to survivors across all interventions, indicating that survivors could carry out the intervention activities after the intervention ended. However, technical problems with video calling affected intervention activity execution somewhat, though the nurse consultant addressed issues promptly (e.g., by having telephone sessions). Most survivors regarded video calling positively for its travel-saving benefits. For all interventions, survivors indicated a sense of fulfilment after completion, especially in health consciousness for the lifestyle and empowerment interventions (Tables 2 and 3):

*"You are confronted with the facts again, and that is good, isn’t it?” (Male, 44 y, participant REVIVER lifestyle intervention)*

##### Integration and implementation

Factors influencing the success and barriers of integration and implementation of the REVIVER interventions, as perceived by survivors, are detailed in Tables 2 and 3. While not unanimous, some lifestyle and empowerment intervention participants felt the overall duration, including session intervals and the number of sessions, was suboptimal. The lack of specific advice for survivors was a barrier to the lifestyle and empowerment interventions. The success factors of all three interventions were mainly related to the qualities of the coaching nurse consultants, such as sincerity, commitment, compassion, accessibility, supportiveness, and openness to feedback. In addition, the nurse consultant’s approach, attitude, and communication skills, including effective questioning, attentive listening and clear communication, were consistently identified as success factors. The flexibility in session scheduling resulting from the person-centred care approach and video calling options was echoed in all interventions as a success factor of the REVIVER interventions. One survivor participating in the empowerment intervention said:

*"At that moment, I really had the idea that actually everything depended on me. It was my input that was needed, and I was asked if I agreed or not [with things], which were possible tips [I could apply], [etc]. So [I had] the feeling that […] she did her best to make it as personal as possible.” (Female, 29 y, participant REVIVER empowerment intervention)*

Survivors appreciated the "no pressure/obligation" approach of person-centred care, allowing them complete control over decisions and time management. Nevertheless, on intervention quality, some wished the nurse consultant had more lifestyle qualifications, as expressed in this quote:

*“It would have been welcome if someone has also done a study on this [lifestyle] or can give some practical tips on some things.” (Female, 29 y, participant REVIVER lifestyle intervention)*

A key success factor for REVIVER interventions was HCPs’ ability to assist survivors after clinic visits. Content-wise, HCPs valued the open and personalised approach of the empowerment intervention, as well as the low participation threshold, as illustrated by the following quote:

*“You know, psychologist sounds ...no offence, but often sounds very heavy and then people think: “No, I don’t want that”. But they do often want this, and then you just notice that it can yield something. I find that very positive." (Female, 63 y, nurse consultant)*

HCPs strongly believed that the REVIVER interventions made survivors feel heard, making this a critical success factor. Concerning the quality of the lifestyle intervention, one of the nurse consultants acknowledged occasional challenges in comprehensive lifestyle knowledge but felt competent enough to deliver all interventions. Implementation facilitators included support from fellow HCPs, organisational backing, and effective video-calling software. Lastly, HCPs perceived the interventions as feasible to implement long-term.

##### Tips for future implementation

The interviews and the focus group also yielded valuable suggestions for future implementation of the REVIVER interventions at other centres or departments (Supplementary Table 3). Tailoring interventions more to survivors' preferences, such as the number of sessions and duration, was recommended. On an organisational level, survivors and HCPs suggested aftercare, like a phone call or video call session after some time to check on the survivor, conducted by a GP/physiotherapist or nurse consultant (Supplementary Table 3).

#### Feasibility evaluation – quantitative assessments

##### Demand

**Fig. 2:**
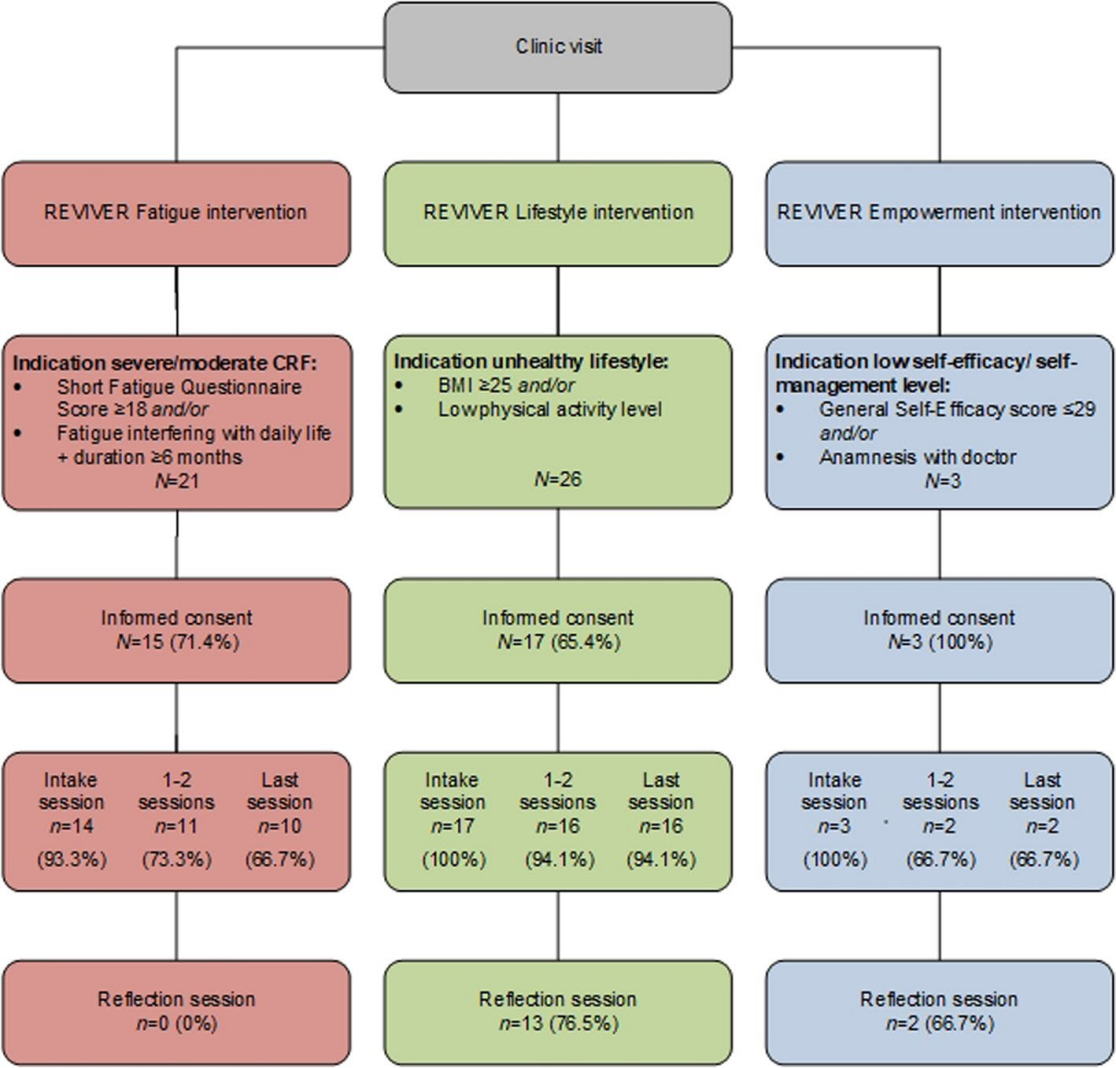
Flowchart of the REVIVER study, including demand and adherence to the interventions

Figure 2 illustrates the participant flow in the REVIVER interventions and the demand for the interventions. Over the 29-month recruitment period, 21, 26 and 3 CAYA cancer survivors were eligible for the fatigue, lifestyle or empowerment intervention, respectively. Meeting the criteria for success regarding demand, 71.4% of eligible participated in the fatigue intervention, and 65.4% participated in the lifestyle intervention (Fig. 2, Supplementary Table 1, Supplementary Table 2). A primary reason for non-participation included a lack of interest in the study measurements. While all eligible survivors participated in the empowerment intervention, the limited invitations (*n* = 3) indicated low demand for this intervention.

##### Adherence

Concerning the adherence of participants to the intervention sessions (Fig. 2, Supplementary Table 2), there were high dropout rates for the fatigue and empowerment intervention (33.3%) after the T0-measurement and intake session. These rates exceeded the criteria of a maximum of 10% dropout rate. The primary reason for dropout in the fatigue intervention was the indication of additional psychological support to address underlying issues such as depression. Nonetheless, adherence to the interventions (i.e., the percentage of survivors adhering to the planned sessions) was high for all interventions (Supplementary Table 3). In the fatigue intervention, no participants engaged in a reflection session due to misinterpreting the study guide.

### Results of potential effectiveness evaluation

#### Potential effectiveness evaluation – quantitative assessments

**Fig. 3:**
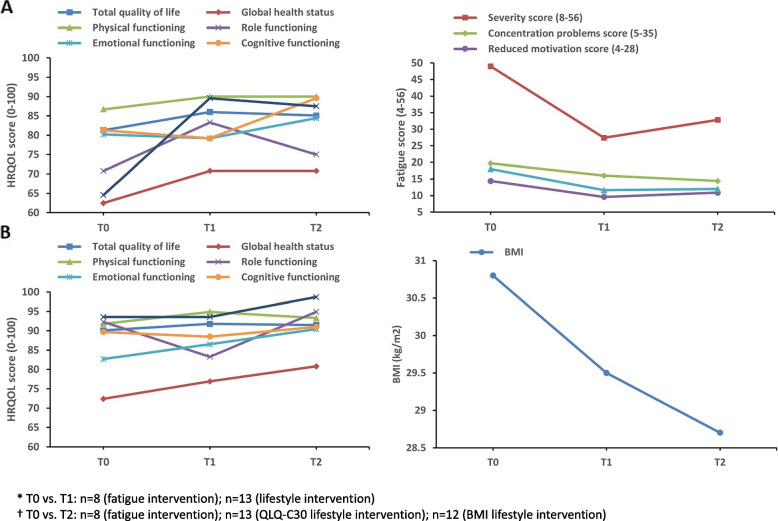
HRQOL, fatigue, and/or BMI of participants of the fatigue (**A**) or lifestyle intervention (**B**)^*,^^†^ ^*^ T0 vs. T1: *n*=8 (fatigue intervention); *n*=13 (lifestyle intervention). ^†^ T0 vs. T2: n=8 (fatigue intervention); *n*=13 (QLQ-C30 lifestyle intervention); *n*=12 (BMI lifestyle intervention)

**Fig. 4:**
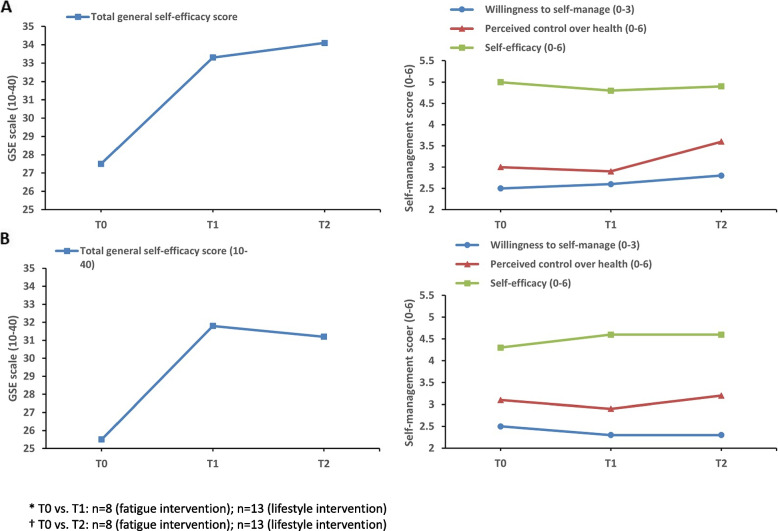
Self-efficacy and self-management (SeMaS) of participants of the fatigue (**A**) or lifestyle intervention (**B**))^*,^^†^ ^*^ T0 vs. T1: *n*=8 (fatigue intervention); *n*=13 (lifestyle intervention). ^†^ T0 vs. T2: *n*=8 (fatigue intervention); *n*=13 (lifestyle intervention)

The findings on potential effectiveness evaluation are presented in Figs. 3 and 4, Supplementary Table 5, and Supplementary Table 6. Statistical effectiveness evaluation of the empowerment intervention was impossible due to the limited number of participants completing the intervention (*n* = 2). Due to missing data at T1 and T2 caused by participants dropping out prematurely or not filling in the questionnaires, only complete pairs (i.e., T0 and T1; T0 and T2) of data of 8 and 13 participants of the fatigue and lifestyle intervention, respectively, were available for effectiveness evaluation with paired t-tests.

##### REVIVER fatigue intervention

Comparing baseline (T0) with post-intervention (T1), the REVIVER fatigue intervention exhibited potential minor effects with paired t-tests for total quality of life (*d* = 0.42) and physical functioning (*d* = 0.42). In contrast, medium effect sizes were observed for global health status (*d* = 0.71), role functioning (*d* = 0.54) and social functioning (*d* = 0.75). The REVIVER fatigue intervention showed more substantial effects comparing T0 with 6 months post-intervention (T2), with large effect sizes observed for cognitive (*d* = 0.84) and social functioning (*d* = 0.92). Participants showed reductions in total fatigue (*d* = -1.34) and the subdomains severity of fatigue (*d* = -1.69) and reduced physical activity (*d* = -1.06) from T0 to T1. These improvements persisted at T2, with large effect sizes observed across all fatigue subdomains. In the fatigue intervention, general self-efficacy scores improved from T0 to T1 (*d* = 0.51) and T0 to T2 (*d* = 0.59). Concerning self-management, in the fatigue intervention, the willingness to self-manage (*d* = 0.54) and the perceived control over health (*d* = 1.06) were improved at T2.

##### REVIVER lifestyle intervention

When evaluating the results of the paired t-test results, it was observed that lifestyle participants had higher HRQOL scores at T0 vs. fatigue intervention participants with minor improvements in total quality of life (*d* = 0.19), global health (*d* = 0.36), physical functioning (*d* = 0.31), and role functioning (*d* = 0.28) at T1. BMI decreased from 30.8 at T0 to 29.5 at T1 (*d* = -0.55) and 28.7 at T2 (*d* = -0.48). Small and medium effect sizes at T1 were seen for physical activity measured by the IPAQ questionnaire in the Leefstijlvragenlijst (*d* = 0.35) and total weekly minutes of physical activity via the SQUASH questionnaire (*d* = 0.52), respectively. However, fewer active minutes were observed at T2 vs. T1. Diet improved at T1 (*d* = -0.65) and T2 (*d* = -0.73), and alcohol scores at T2 (*d* = -0.50). General self-efficacy scores increased post-intervention (*d* = 0.60) and slightly decreased at 6-month follow-up (*d* = 0.59). In lifestyle intervention participants, a positive trend towards higher self-efficacy, as assessed by the SeMaS questionnaire, was observed (*d* = 0.73).

### Results fidelity

REVIVER intervention fidelity, assessed via nurse consultants’ reports, participant interviews and the HCPs focus group (Supplementary Table 4), showed nearly all components were executed as planned. The fatigue intervention lacked a 6-month post-intervention reflection session due to unclearness in the intervention manual. Intake sessions for all interventions exceeded planned times (45–60 min vs. 30–45 min), while coaching sessions were shorter (15–30 min vs. 30–45 min) for the fatigue and lifestyle intervention. The reflection sessions for the lifestyle and empowerment intervention were also shorter than planned (i.e., 15–30 min vs. 30–45 min). The lifestyle intervention was primarily completed with 2 coaching sessions instead of 3–6, and it was completed within 3 months, suggesting a higher session frequency (Supplementary Table 4).

## Discussion

This mixed-methods study demonstrates the feasibility and potential effectiveness of the REVIVER nurse-led video-coaching fatigue and lifestyle interventions. Results indicate enhanced self-efficacy and self-management with the REVIVER fatigue and lifestyle interventions. The interventions are deemed acceptable, practical and successfully integratable. However, lower feasibility for the REVIVER empowerment intervention due to reduced demand and relatively high dropout rates in the fatigue and empowerment interventions were observed. Challenges to sustainability, technical issues and the short duration of the REVIVER lifestyle and empowerment intervention were identified as barriers. Notably, this is the first feasibility study on assessing the feasibility and potential effectiveness of the REVIVER interventions. Utilising mixed methods, our study provides a comprehensive and in-depth understanding of the factors influencing feasibility outcomes regarding demand, adherence, acceptability, practicality, integration/implementation, and potential effectiveness.

The findings of this study indicate that all interventions were generally perceived as moderately good (empowerment intervention) or good (fatigue and lifestyle intervention), with all interventions being recommended to others. Some participants of the lifestyle and empowerment intervention needed additional (psychosocial) support after completion, possibly due to nurse consultants lacking in-depth knowledge of health behaviours and psychosocial issues. This aligns with prior research highlighting a lack of health behaviour knowledge among HCPs affiliated with survivorship care clinics across Europe [59]. Furthermore, in another published paper, it has been indicated as a barrier for childhood cancer survivors to adopt or maintain healthy behaviours [60]. Therefore, additional training in health behaviours could benefit the lifestyle intervention. Considering the relevance and potential impact on outcomes, a discussion on whether nurse consultants should receive more education in psychosocial issues is warranted. Notably, nurse consultants can quickly consult with a psychologist during multidisciplinary psychosocial meetings and one-on-one intervision sessions, facilitating communication of comfort levels and boundary establishment when addressing psychosocial issues in interventions.

In accordance with a study by Post et al. among breast cancer survivors on web-based survivorship interventions, the utilisation of video calling as a mode of delivery was deemed acceptable by many survivors due to reduced travel burden. Moreover, HCPs were open towards to future use [37]. Some survivors preferred (partial) face-to-face sessions due to a perceived lack of personal connection online, which is potentially solvable by prior in-person meetings. Other barriers to video calling included technical difficulties, but a stable software program could enhance seamless video calling despite inevitable internet challenges.

In feasibility studies, ensuring the sustainability of implemented interventions is imperative and requires assessing participant’s ability to carry out activities effectively. Consequently, establishing a person-centred care foundation, emphasising assessing readiness, importance, confidence, and knowledge among participants before initiating the intervention, is crucial. This proactive approach helps mitigate potential challenges related to executing these activities. Notably, fatigue and lifestyle intervention participants faced occasional setbacks in their progress post-intervention, which could be resolved by arranging aftercare once the interventions have concluded. The desire for aftercare (e.g., by the nurse consultant, GP, or physiotherapist), supported by participants and HCPs, emphasises its added value across all interventions, ensuring continued support beyond the intervention.

Though participants were predominantly positive about the interventions, the lifestyle and empowerment intervention could improve by incorporating more personalisation in session number and duration of the interventions and individual sessions. Participant self-management abilities and self-efficacy levels are pivotal in determining the optimal balance in duration and frequency. Acknowledging diverse survivor needs is crucial: some may only need guidance, while others require more coaching in stimulating self-management and self-efficacy. Therefore, the coaching nurse consultant must assess these factors during the intervention.

The findings also highlight several success factors for effectively integrating and implementing the REVIVER interventions. For instance, nurse consultant qualities, especially familiarity with person-centred care, were pivotal in facilitating the successful adaption of behaviours. The presence of a nurse consultant who is familiar with person-centred care maintained a close relationship with the survivor, possessing an empathetic understanding of the unique challenges that CAYA cancer survivors face, and being open to listening created a supportive environment without any obligation connections. This may increase the self-willingness and motivation of survivors to change their behaviours.

For the REVIVER fatigue intervention, based on CBT, there was a substantial demand (71.4%) potentially driven by the high prevalence of CRF in the (childhood) cancer survivor population (26.1%) and its adverse effects on their daily lives could elucidate their motivation for participation, [19]. While direct comparisons are difficult due to differences in the study population, intervention and recruitment strategies, this demand for the REVIVER fatigue intervention surpassed that reported in earlier research by Esser et al. (42%), Gielissen et al. (67%) and Jim et al. (59%) [28, 61, 62]. For the REVIVER lifestyle intervention, the demand was slightly lower (65.4%) than that for a physical activity intervention studied by Devine et al. among CAYA cancer survivors (88%) [36]. Considering that participants of this intervention need to make new healthy routines to improve lifestyle behaviours on their own, good timing and readiness for participation in this intervention is crucial. This aligns with what was mentioned by HCPs in this study. Unlike the other REVIVER interventions, the empowerment intervention had only three eligible survivors. HCPs used the GSE scale with a < 29 cut-off point, derived from the mean score in German cancer patients [63]. This threshold may have been too high for CAYA cancer survivors who, having moved beyond active treatment, generally exhibited higher baseline self-efficacy. Additionally, the broad nature of the empowerment intervention might have led to low demand, possibly due to survivors not fully understanding its scope. Future adaptations in eligibility assessment are necessary for the empowerment intervention.

Despite high demand, a substantial proportion of participants did not complete the fatigue intervention (33.3%), surpassing the targeted threshold of 10% and rates reported in prior research [28, 29, 61, 62]. This was predominantly attributed to underlying issues (e.g., depression, stress, etc.) requiring more specialised (psychosocial) care. As this study served as a pilot, future eligibility assessments for fatigue intervention should include comprehensive screening for factors like anxiety and depression. The dropout rate in the empowerment intervention also exceeded 10%, but this can be explained by low demand.

The REVIVER fatigue and lifestyle intervention, though based on a feasibility study with only 35 participants, showed promising results regarding potential effectiveness. The observed improvements in fatigue, with a medium effect size in role functioning post-intervention, are clinically significant for survivors, enhancing their daily lives and quality of life. The observed improvements in fatigue, with a medium effect size in role functioning post-intervention, are clinically significant for survivors, enhancing their daily lives and overall quality of life. The lifestyle intervention, primarily relying on motivational interviewing, also showed medium effect sizes post-intervention, suggesting positive changes in health behaviours like diet and BMI. Although there was a partial regression in BMI improvement at the 6-month follow-up, a substantial portion of survivors improved their diet, denoting a new dietary pattern. Self-efficacy, fostered by incorporated person-centred care principles throughout regular clinic visits of survivors and the intervention, increased in both interventions, encouraging the continuation of new positive behavioural patterns. Furthermore, effects persisted even higher at the 6-month follow-up, indicating survivors’ commitment to intervention activities and maintenance of beneficial behaviours. This contrasts with previous studies on fatigue and lifestyle interventions, showing less enduring effects beyond follow-up periods [61, 64]. In upcoming research conducted by Bouwman et al., the investigation will seek to determine whether a lifestyle intervention based on person-centred care principles may yield potential impacts on health behaviours, both in the short-term (i.e., directly following the intervention) and in the longer term (i.e., 4 months post-intervention) [65]. Exploring sustained effects beyond 4 or 6 months would also be interesting in future studies.

The utilisation of a mixed methods design, using both qualitative and quantitative measures across three measurement points, contributes to the robustness of the results of this study by methodological triangulation as it provides a comprehensive and in-depth insight into the feasibility and potential effectiveness of the personalised REVIVER interventions. However, the small number of participants in the empowerment intervention limits statistical analysis. In addition, due to the exploratory nature of this feasibility study and limited sample sizes, mixed effect modelling, as proposed in the protocol paper, was unattainable. The focus shifted toward the potential effect sizes, and *P*-values of < 0.05 were considered as only potentially significant. In addition, the study's personalised nature presented strengths and challenges, particularly for the lifestyle intervention, with tailored strategies as a strength and the complexities of individual objectives as a limitation. Another limitation of this study is the missing data from dropouts or uncompleted questionnaires, potentially distorting the potential effectiveness outcomes and limiting the transferability of the results. Lastly, the self-reported data from questionnaires increases the risk of social desirability bias and subjective interpretation and may influence the outcomes.

## Conclusions

Overall, our study shows that the REVIVER fatigue and lifestyle interventions, despite a lower adherence to the fatigue intervention, are feasible for further implementation and dissemination. Feasibility for the empowerment intervention was lower due to a decreased demand, and dropout rates were relatively high for the fatigue and empowerment interventions. Moreover, the fatigue and lifestyle interventions show potential effectiveness with medium and large effect sizes for HRQOL, fatigue, lifestyle, self-efficacy, and self-management outcomes. The medium and high effect sizes found in this study not only hold potential clinical significance, but also serve as valuable input for future studies investigating the efficacy of the REVIVER interventions.

### Supplementary Information

Supplementary Material 1.

Supplementary Material 2.

Supplementary Material 3.

Supplementary material 4.

Supplementary Material 5.

Supplementary material 6.

## Data Availability

The datasets used and/or analysed during the current study are available from the corresponding author upon reasonable request.

## Abbreviations

AUDIT: Alcohol Use Disorders Identification Tests
BMI: Body Mass Index
CAYA: Childhood Adolescent Young Adult
CBT: Cognitive Behavioural Therapy
CIS: Checklist Individual Strength
CNS: Central Nervous System
CRF: Cancer Related Fatigue
eHealth: Electronic Health
GP: General Practitioner
GSE: General Self-Efficacy
HCP: Healthcare Professional
HRQOL: Health-Related Quality of Life
IPAQ: International Physical Activity Questionnaire
IQR: Interquartile Range
REVIVER: NuRse-led WEb_based Video-coaching InterVEntions for SuRvivors
SeMaS: Self-Management Screening
SQUASH: Short Questionnaire to Assess Health-enhancing physical activity
