# Supplementary material for: Feasibility and potential effectiveness of nurse-led video-coaching interventions for childhood, adolescent, and young adult cancer survivors: the REVIVER study
Source: BMC Cancer. 2024 Jun 11;24:722. doi: 10.1186/s12885-024-12430-3 (PMC11167751; doi:10.1186/s12885-024-12430-3)
Supplement: Supplementary file 1 — Supplementary Material 1. [file 12885_2024_12430_MOESM1_ESM.docx]

**Supplementary Table 1.** Assessment overview of success criteria of feasibility outcomes of the REVIVER study

| **Feasibility outcome** | **Assessment** | **Success criteria** |
| --- | --- | --- |
| Acceptability | Evaluated with a qualitative interview:  1. Rating (scale 0-10) overall intervention (survivors)  2. Rating (scale 0-10) overall intervention (HCPs)  3. Rating (scale 0-10) on willingness to recommend intervention to other survivors (survivor | 1. Mean overall rating intervention (survivors): ≥5  2. Mean overall score intervention (coaches): ≥5  3. Mean score “I would recommend the intervention to other survivors” (survivors): ≥5 |
| Demand | Evaluated with registration forms:  1. Percentage of eligible survivors who agree to participate in one of the REVIVER interventions and study | 1. Percentage of eligible survivors who agree to participate in one of the REVIVER interventions and study ≥25% |
| Adherence to intervention | Evaluated with the nurses’ reports:  1. Percentage of dropouts (survivors)  2. Percentage of participants following the sessions according to plan (survivors) | 1. Percentage of dropouts ≤10%  2. Percentage of participants following the sessions according to plan ≥80% |
