# Supplementary material for: Feasibility and potential effectiveness of nurse-led video-coaching interventions for childhood, adolescent, and young adult cancer survivors: the REVIVER study
Source: BMC Cancer. 2024 Jun 11;24:722. doi: 10.1186/s12885-024-12430-3 (PMC11167751; doi:10.1186/s12885-024-12430-3)
Supplement: Supplementary file 2 — Supplementary Material 2. [file 12885_2024_12430_MOESM2_ESM.docx]

**Supplementary Table 2.** Inclusion/measurement completion rates participants REVIVER study in total and per intervention

|  | **Fatigue** | **Lifestyle** | **Empowerment** |
| --- | --- | --- | --- |
| **Inclusion intervention** |  |  |  |
| Nr. invited survivors | 21 | 26 | 3 |
| Nr. Included survivors (participation rate (%)) | 15 (71.4%) | 17 (65.4%) | 3 (100%) |
| Nr. included survivors (nr. included survivors/ expected inclusion rate) | 15 (75%) | 17 (85%) | 3 (15%) |
| Nr. non-responders (nr. non responders/ nr. invited survivors) | 2 (9.5%) | 4 (15.4%) | 0 (0%) |
| Nr. refusals (nr. refusals/ nr. invited survivors) | 3 (14.13%) | 5 (19.2%) | 0 (0%) |
| Nr. exclusions (nr. exclusions/ nr. invited survivors) | 1 (4.8%) | 0 (0%) | 0 (0%) |
| **Completion intervention** |  |  |  |
| Nr. included survivors completing the intervention | 10 (66.7%) | 16 (94.1%) | 2 (66.7%) |
| Nr. dropouts (nr. dropouts/ nr. included survivors) | 5 (33.3%) | 1 (3.8%) | 1 (33.3%) |
| **Assessments** |  |  |  |
| Nr. included survivors completing ≥2/3 assessments | 10 (66.7%) | 15 (88.2%) | 2 (66.7%) |
| **Adherence to intervention sessions**^*^ |  |  |  |
| Nr. joined intake sessions (nr. joined intake sessions/ nr. intake sessions planned) | 15 (100%) | 17 (100%) | 3 (100%) |
| Nr. joined regular sessions (nr. joined regular sessions/ nr. regular sessions planned) | 25 (100%) | 32 (96.9%) | 5 (100%) |
| Nr. joined closing sessions (nr. joined closing sessions/ nr. closing sessions planned) | 10 (100%) | 13 (92.9%) | 2 (100%) |
| Total nr. joined intervention sessions (nr. joined sessions/ nr. sessions planned) | 50 (100%) | 62 (96.9%) | 10 (100%) |

^*^ Determined by comparing the sessions planned vs. the actual joined sessions
