# Supplementary material for: Feasibility and potential effectiveness of nurse-led video-coaching interventions for childhood, adolescent, and young adult cancer survivors: the REVIVER study
Source: BMC Cancer. 2024 Jun 11;24:722. doi: 10.1186/s12885-024-12430-3 (PMC11167751; doi:10.1186/s12885-024-12430-3)
Supplement: Supplementary file 3 — Supplementary Material 3. [file 12885_2024_12430_MOESM3_ESM.docx]

**Supplementary Table 3.** Tips for future implementation of the REVIVER interventions provided by survivors and HCPs

| **Level** | **Tip** |
| --- | --- |
| **Survivors** |  |
| Intervention level | - Having a private space for intervention |
|  | - Participating survivors should take time for intervention - Providing more options for personalization |
|  | - Advertise intervention for survivors |
|  | - Better preparation nurse for intervention |
| Organisation level | - Providing aftercare after intervention |
|  | - Arranging reimbursement intervention |
| **HCPs** |  |
| Intervention level | - Personalize intervention to survivor |
|  | - Focus on contextual factors survivors with intervention |
|  | - Provide intervention at right timing for survivor |
|  | - Discuss participation with survivor in a later stage when timing is not right |
|  | - Provide a feedback letter with outcomes intervention for GP/referrer |
|  | - Use an user friendly software application |
|  | - Use a telephone in case of technical problems |
| Organisation level | - Provision of aftercare by GP/physiotherapist |
|  | - Provision of aftercare by nurse |
|  | - Delivery interventions by a nurse |
|  | - Have enough funding/resources |
|  | - Provision by a skilled nurse |
|  | - Personalize approach implementation to department/centre |
