# Supplementary material for: Feasibility and potential effectiveness of nurse-led video-coaching interventions for childhood, adolescent, and young adult cancer survivors: the REVIVER study
Source: BMC Cancer. 2024 Jun 11;24:722. doi: 10.1186/s12885-024-12430-3 (PMC11167751; doi:10.1186/s12885-024-12430-3)
Supplement: Supplementary file 4 — Supplementary material 4. [file 12885_2024_12430_MOESM4_ESM.docx]

**Supplementary Table 4.** Fidelity assessment of the REVIVER interventions

| **Element of intervention fidelity** | **General process question** | **Data source and data collection method** | **Fatigue intervention**  **(component/measure + fidelity score)^*†^** | | **Lifestyle intervention**  **(component/measure + fidelity score)^*†^** | | **Empowerment intervention**  **(component/measure + fidelity score)^*†^** | |
| --- | --- | --- | --- | --- | --- | --- | --- | --- |
| Content | Was each of the intervention components implemented as planned? | - Reports of nurses (quantitative) - Interviews with participants (qualitative) - Focus group with healthcare professionals as stakeholders (qualitative) | *Recruitment process*  *Intake session*  *Coaching sessions*  *Reflection/last session*  *Module setting*  *Video calling*  *Person-centred approach*  *Assignments/tips provision by nurse*  *Reports by nurse*  *Multidisciplinary team* | +  +  +  -  +/-  +/-  +  +  +  + | *Recruitment process*  *Intake session*  *Coaching sessions*  *Reflection/last session*  *Goal setting*  *Video calling*  *Person-centred approach*  *Motivational interviewing*  *Assignments/tips provision by nurse*  *Reports by nurse*  *Multidisciplinary team* | +  +  +  +/-  +/-  +/-  +/-  +  +/-  +  + | *Recruitment process*  *Intake session*  *Coaching sessions*  *Reflection/last session*  *Goal setting*  *Video calling*  *Person-centred approach*  *Motivational interviewing*  *Assignments/tips provision by nurse*  *Reports by nurse*  *Multidisciplinary team* | +  +  +  +/-  +/-  +/-  +/-  +  +  +  + |
| Frequency/duration (dosage, dose delivery) | Were the intervention components implemented as often and for as long as planned? | - Reports of nurses | *Frequency intake session*   - **100%**   *Frequency coaching sessions*   - 1 session: 0% - 2 sessions: 10% - **3 sessions: 90%**   *Frequency reflection session*   - 0%   *Duration intake sessions:*   - 10-15 min: 0% - **30-45 min: 26.7%** - 45-60 min: 73.3% - 60-75 min: 0%   *Duration coaching sessions:*   - 10-15 min: 8.6% - 15-30 min: 60% - **30-45 min: 28.6%** - 45-60 min: 2.9%   *Duration reflection sessions:*   - N/A   *Time frame of intervention:*   - **≤3 months: 60%** - >3 months: 40% |  | *Frequency intake session*   - **100%**   *Frequency coaching sessions*   - 1 session: 6.3% - 2 sessions: 68.8% - **3 sessions: 25%**   *Frequency reflection session*   - 100%   *Duration intake sessions:*   - 10-15 min: 0% - **30-45 min: 29.4%** - 45-60 min: 64.7% - 60-75 min: 5.9%   *Duration coaching sessions:*   - 10-15 min: 8.6% - 15-30 min: 65.7% - **30-45 min: 22.9%** - 45-60 min: 2.9%   *Duration reflection sessions:*   - 10-15 min: 0% - 15-30 min: 84.6% - **30-45 min: 15.4%** - 45-60 min: 0%   *Time frame of intervention:*   - **≤3 months: 73.3%** - >3 months: 26.7% |  | *Frequency intake session*   - **100%**   *Frequency coaching sessions*   - 1 session: 0% - 2 sessions: 0% - **3 sessions: 100%**   *Frequency reflection session*   - 0%   *Duration intake sessions:*   - 10-15 min: 0% - **30-45 min: 0%** - 45-60 min: 100% - 60-75 min: 0%   *Duration coaching sessions:*   - 10-15 min: 0% - 15-30 min: 25% - **30-45 min: 75%** - 45-60 min: 0%   *Duration reflection sessions:*   - 10-15 min: 0% - 15-30 min: 100% - **30-45 min: 0%** - 45-60 min: 0%   *Time frame of intervention:*   - **≤3 months: 0%** - >3 months: 100% |  |

^*^ + = fidelity is met; +/-: fidelity is met to a large extent; -: fidelity is not met

**^†^** In bold depicted are the frequencies, durations or time frame originally planned for the REVIVER interventions
