# Supplementary material for: Feasibility and potential effectiveness of nurse-led video-coaching interventions for childhood, adolescent, and young adult cancer survivors: the REVIVER study
Source: BMC Cancer. 2024 Jun 11;24:722. doi: 10.1186/s12885-024-12430-3 (PMC11167751; doi:10.1186/s12885-024-12430-3)
Supplement: Supplementary file 5 — Supplementary Material 5. [file 12885_2024_12430_MOESM5_ESM.docx]

**Supplementary Table 5.** Results pre-intervention (T0), post-intervention (T1) and at 6-months follow-up (T2) of the REVIVER fatigue intervention^*†^

|  | Mean (SD) | | *P*-value | Cohen’s *d*  effect size | Mean (SD) | | *P*-value | Cohen’s *d*  effect size |
| --- | --- | --- | --- | --- | --- | --- | --- | --- |
|  | T0 | T1 |  |  | T0 | T2 |  |  |
| **Quality of life (QLQ_C30) (n=8)** |  |  |  |  |  |  |  |  |
| Total quality of life score (0-100) | 81.3 (9.7) | 86.0 (8.3) | 0.269 | 0.42 | 80.0 (8.4) | 85.1 (7.9) | 0.104 | **0.66** |
| Global health status (0-100) | 62.5 (16.7) | 70.8 (17.3) | 0.086 | **0.71** | 64.6 (15.9) | 70.8 (12.6) | 0.244 | 0.45 |
| Physical functioning (0-100) | 86.7 (13.3) | 90.0 (9.4) | 0.275 | 0.42 | 83.3 (12.8) | 90.0 (9.4) | 0.086 | **0.71** |
| Role functioning (0-100) | 70.8 (26.4) | 83.3 (17.8) | 0.170 | **0.54** | 70.8 (26.4) | 75.0 (19.9) | 0.598 | 0.20 |
| Emotional functioning (0-100) | 80.2 (14.7) | 79.2 (11.8) | 0.732 | 0.13 | 82.3 (13.7) | 84.4 (12.9) | 0.563 | 0.22 |
| Cognitive functioning (0-100) | 81.3 (16.5) | 79.2 (19.4) | 0.785 | -0.10 | 79.2 (14.8) | 89.6 (12.4) | **0.049** | **0.84** |
| Social functioning (0-100) | 64.6 (33.9) | 89.6 (15.3) | 0.072 | **0.75** | 60.4 (30.8) | 87.5 (14.8) | **0.035** | **0.92** |
| **Fatigue (CIS20) (n=7)** |  |  |  |  |  |  |  |  |
| Total fatigue score (20-140) | 100.4 (14.6) | 63.4 (25.8) | **0.012** | **-1.34** | 95.9 (18.1) | 68.9 (13.8) | **0.003** | **-1.58** |
| Severity score (8-56) | 49.0 (5.2) | 27.4 (11.0) | **0.004** | **-1.69** | 46.1 (8.0) | 32.8 (5.8) | **0.005** | **-1.40** |
| Concentration problems score (5-35) | 19.7 (8.2) | 16.0 (6.9) | 0.096 | **-0.74** | 19.1 (7.5) | 14.4 (4.9) | **0.033** | **-0.93** |
| Reduced motivation score (4-28) | 14.4 (5.2) | 9.6 (5.4) | 0.130 | **-0.66** | 14.1 (5.8) | 10.9 (4.0) | **0.016** | **-1.12** |
| Reduced physical activity score (3-21) | 18.0 (2.7) | 11.6 (5.1) | **0.031** | **-1.06** | 17.5 (3.1) | 12.0 (4.0) | **0.012** | **-1.20** |
| **Lifestyle (Leefstijlvragenlijst) (n=8)** |  |  |  |  |  |  |  |  |
| Physical activity score (0-315) | 54.3 (26.9) | 57.4 (34.7) | 0.790 | 0.10 | 53.3 (27.9) | 51.8 (30.2) | 0.920 | -0.13 |
| Diet score (3-9) | 6.4 (1.5) | 6.3 (0.9) | 0.732 | -0.13 | 5.9 (1.1) | 6.8 (1.0) | 0.064 | **0.77** |
| BMI | 28.7 (4.8) | 27.7 (5.1) | 0.122 | **-0.62** | 28.8 (4.4) | 30.1 (6.5) | 0.308 | 0.42 |
| Alcohol score (0-40) | 1.4 (1.7) | 1.1 (1.5) | 0.451 | -0.28 | 1.1 (1.5) | 1.1 (1.5) | 1.000 | 0.00 |
| **Physical activity (SQUASH) (n=8)** |  |  |  |  |  |  |  |  |
| Total minutes per week active | 1639 (664) | 1664 (776) | 0.925 | 0.04 | 1702 (557) | 1587 (1195) | 0.801 | -0.09 |
| Total activity score | 4318 (2016) | 4449 (2359) | 0.851 | 0.07 | 4473 (1840) | 4236 (3305) | 0.868 | -0.06 |
| **Self-efficacy (GSE Scale) (n=8)** |  |  |  |  |  |  |  |  |
| Total general self-efficacy score (10-40) | 27.5 (11.1) | 33.3 (4.7) | 0.196 | **0.51** | 28.1 (11.1) | 34.1 (4.3) | 0.141 | **0.59** |
| **Self-management (SeMaS) (n=8)** |  |  |  |  |  |  |  |  |
| Willingness to self-manage (0-3) | 2.5 (0.5) | 2.6 (0.5) | 0.598 | 0.20 | 2.5 (0.5) | 2.8 (0.5) | 0.170 | **0.54** |
| Perceived control over health (0-6) | 3 (1.3) | 2.9 (1.2) | 0.685 | -0.15 | 2.9 (1.2) | 3.6 (1.4) | **0.020** | **1.06** |
| Self-efficacy (0-6) | 5 (1.2) | 4.8 (1.0) | 0.563 | -0.22 | 5.1 (1.1) | 4.9 (1.3) | 0.563 | -0.22 |

^*^ Paired t-test results

^†^ Displayed in bold are *P*-values <0.05 and Cohen’s *d* effect sizes >0.5 representing medium or large effect sizes
