# Supplementary material for: Feasibility and potential effectiveness of nurse-led video-coaching interventions for childhood, adolescent, and young adult cancer survivors: the REVIVER study
Source: BMC Cancer. 2024 Jun 11;24:722. doi: 10.1186/s12885-024-12430-3 (PMC11167751; doi:10.1186/s12885-024-12430-3)
Supplement: Supplementary file 6 — Supplementary material 6. [file 12885_2024_12430_MOESM6_ESM.docx]

**Supplementary Table 6.** Results pre-intervention (T0), post-intervention (T1) and at 6-months follow-up (T2) of the REVIVER lifestyle intervention^*†^

|  | Mean (SD) | | *P*-value | Cohen’s *d*  effect size | Mean (SD) | | *P*-value | Cohen’s *d*  effect size |
| --- | --- | --- | --- | --- | --- | --- | --- | --- |
|  | T0 | T1 |  |  | T0 | T2 |  |  |
| **Quality of life (QLQ_C30) (n=13)** |  |  |  |  |  |  |  |  |
| Total quality of life score (0-100) | 90.1 (11.6) | 91.8 (10.7) | 0.431 | 0.19 | 89.8 (11.5) | 91.5 (10.9) | 0.196 | 0.38 |
| Global health status (0-100) | 72.4 (14.6) | 76.9 (16.4) | 0.222 | 0.36 | 72.4 (14.6) | 80.8 (12.9) | **0.047** | **0.61** |
| Physical functioning (0-100) | 91.8 (15.2) | 94.9 (12.5) | 0.337 | 0.31 | 91.8 (15.2) | 93.3 (11.5) | 0.584 | 0.16 |
| Role functioning (0-100) | 92.3 (20.0) | 83.3 (17.8) | 0.170 | 0.28 | 91.0 (20.0) | 94.9 (14.2) | 0.082 | 0.53 |
| Emotional functioning (0-100) | 82.7 (18.8) | 86.5 (16.9) | 0.502 | 0.13 | 80.8 (18.1) | 90.4 (12.7) | **0.033** | **0.67** |
| Cognitive functioning (0-100) | 89.7 (18.7) | 88.5 (21.9) | 0.819 | -0.07 | 89.7 (18.7) | 91.0 (18.8) | 0.808 | 0.07 |
| Social functioning (0-100) | 93.6 (8.4) | 93.6 (12.8) | 0.072 | 0.00 | 94.9 (8.0) | 98.7 (4.6) | **0.082** | **0.53** |
| **Fatigue (CIS20) (n=13)** |  |  |  |  |  |  |  |  |
| Total fatigue score (20-140) | 61.8 (23.3) | 57.5 (23.3) | 0.289 | -0.31 | 63.2 (21.4) | 53.3 (18.6) | **0.032** | **-0.67** |
| Severity score (8-56) | 27.3 (13.0) | 23.9 (12.3) | 0.057 | **-0.58** | 28.2 (11.9 | 23.9 (10.4) | 0.083 | **-0.53** |
| Concentration problems score (5-35) | 13.9 (6.0) | 13.8 (6.1) | 0.896 | -0.04 | 14.0 (5.9) | 12.2 (5.3) | 0.191 | -0.40 |
| Reduced motivation score (4-28) | 9.2 (4.7) | 9.5 (3.9) | 0.651 | 0.13 | 9.9 (4.4) | 8.8 (4.2) | 0.248 | -0.34 |
| Reduced physical activity score (3-21) | 11.4 (4.8) | 10.3 (5.1) | 0.457 | -0.21 | 11.9 (4.2) | 8.5 (3.6) | **0.035** | **-0.66** |
| **Lifestyle (Leefstijlvragenlijst) (n=13)** |  |  |  |  |  |  |  |  |
| Physical activity score (0-315) | 57.4 (36.9) | 83.1 (63.1) | 0.279 | 0.35 | 57.8 (35.3) | 86.9 (70.1) | 0.217 | 0.38 |
| Diet score (3-9) | 6.7 (1.5) | 5.7 (1.4) | 0.058 | **-0.65** | 6.8 (1.4) | 5.5 (1.2) | **0.028** | **-0.73** |
| BMI | 30.8 (5.7) | 29.5 (5.4) | 0.084 | **-0.55** | 30.2 (5.7) | 28.7 (4.1) | 0.110 | -0.48 |
| Alcohol score (0-40) | 5.2 (5.5) | 5.1 (5.8) | 0.724 | -0.11 | 5.4 (5.3) | 4.5 (5.5) | 0.111 | **-0.50** |
| **Physical activity (SQUASH) (n=12)** |  |  |  |  |  |  |  |  |
| Total minutes per week active | 1301 (726) | 2010 (1282) | 0.101 | **0.52** | 1322 (699) | 1448 (700.5) | 0.589 | 0.15 |
| Total activity score | 3518 (1888) | 5741 (4175) | 0.124 | 0.48 | 3680 (1901) | 4107 (2068) | 0.584 | 0.16 |
| **Self-efficacy (GSE Scale) (n=13)** |  |  |  |  |  |  |  |  |
| Total general self-efficacy score (10-40) | 25.5 (11.4) | 31.8 (5.9) | 0.052 | **0.60** | 25.4 (11.4) | 31.2 (4.4) | 0.053 | **0.59** |
| **Self-management (SeMaS) (n=13)** |  |  |  |  |  |  |  |  |
| Willingness to self-manage (0-3) | 2.5 (0.7) | 2.3 (0.6) | 0.337 | -0.28 | 2.4 (0.7) | 2.3 (0.6) | 0.673 | -0.12 |
| Perceived control over health (0-6) | 3.1 (0.8) | 2.9 (0.6) | 0.273 | -0.32 | 2.9 (0.8) | 3.2 (0.4) | 0.219 | 0.36 |
| Self-efficacy (0-6) | 4.3 (1.2) | 4.6 (1.1) | 0.209 | 0.37 | 3.9 (0.9) | 4.6 (1.0) | **0.022** | **0.73** |

^*^ Paired t-test results

^†^ Displayed in bold are *P*-values <0.05 and Cohen’s *d* effect sizes >0.5 representing medium or large effect sizes
